# Bullying Victimization is Associated with Heightened Rates of Anxiety and Depression Among Autistic and ADHD Youth: National Survey of Children’s Health 2016–2020

**DOI:** 10.1007/s10803-024-06479-z

**Published:** 2024-07-22

**Authors:** Amy L. Accardo, Leslie C. Neely, Nancy M. H. Pontes, Manuel C. F. Pontes

**Affiliations:** 1 College of Education, Rowan University, 201 Mullica Hill Road, Glassboro, NJ 08028 USA; 2 Department of Educational Psychology, The University of Texas at San Antonio, 501 W. Cesar E. Chavez Blvd, San Antonio, TX 78207 USA; 3 School of Nursing, Rutgers University, 530 Federal Street, Camden, NJ 08102 USA; 4 Rohrer College of Business, Rowan University, 201 Mullica Hill Road, Glassboro, NJ 08028 USA

**Keywords:** Autism, Bullying, Anxiety, Depression, ADHD, Youth

## Abstract

Autistic youth and youth with ADHD have heightened rates of bullying victimization, anxiety, and depression. The purpose of this research is to use nationally representative US data to 1) estimate the prevalence of anxiety and depression among bullied neurodivergent youth and 2) investigate whether the association between bullying victimization and anxiety or depression is significantly greater among autistic youth and youth with ADHD. For this research, we used five years of data (2016–2020) from the nationally representative National Survey of Children’s Health (NSCH), youth ages 12–17 years (n = 71,973). Data were analyzed with R and the R survey package to estimate average marginal percentages, risk differences, and additive interactions as recommended by STROBE guidelines. The study identified heightened anxiety and depression among bullied autistic or ADHD youth. Results also showed that the increase in the rate of anxiety or depression associated with bullying victimization was significantly greater among autistic youth and youth with ADHD relative to non-autistic non-ADHD youth; interactions were significant among both male and female youth. Autistic youth, youth with ADHD, and youth with co-occurring autism and ADHD are particularly vulnerable to bullying victimization and associated depression and anxiety. Future research is needed to understand why the association between bullying victimization and depression/anxiety is significantly greater among autistic and non-autistic ADHD youth. Recommendations include exploring school-wide anti-stigma initiatives to stop the reciprocal bullying–anxiety/depression cycle, routine bullying and mental health screening of autistic and ADHD youth, and clinical management of bullied autistic and ADHD youth with anxiety or depression.

Victims of bullying experience repetitive, unwanted physical, verbal, relational and/or cyber aggression by another person or group exerting power (Bradshaw et al., 2015; Gladden et al., 2014). Of concern, the US Centers for Disease Control and Prevention (CDC) (2021) report bullying victimization as most prevalent among school age youth, with the highest rates found in middle (28%) and high (16%) schools. The CDC (2021) refer to bullying as youth violence and describe it as widespread with negative consequences including physical, psychological, social, and/or educational harm.

Specifically, bullying victimization among youth has been associated with an increase in delinquency (Zhang et al., 2022), substance abuse (Pontes et al., 2022), violence (Ttofi et al., 2012), and a decline in academic performance (Laith & Vaillancourt, 2022). Additionally, bullying victimization has been associated with negative mental health consequences, including anxiety, depression, self-harm, and suicidality (CDC, 2021), lowered self-esteem (Becker et al., 2017), and negative identity development (Shmulsky et al., 2021). Associations among bullying and mental health are a concern specifically to youth aged 12–17, with the CDC (2022) reporting depression and anxiety highest among youth aged 12–17, and youth of the same age reporting feelings of hopelessness (37%), suicidal ideation (19%) and the formation of a suicide plan (16%) within the last year (2018–19 data).

Bullying victimization among school age youth is a problem recognized worldwide (Laith & Vaillancourt, 2022). In the US, the reported prevalence of middle and high school youth experiencing bullying victimization is commonly reported to be 20–40% (e.g., CDC, 2021). Unfortunately, incidences of bullying are reported at even higher rates for youth with disabilities compared to their nondisabled peers across K-12 grade levels (e.g., Bear et al., 2015; Gage et al., 2021; Malecki et al., 2020), with autistic youth and/or youth with ADHD at an even heightened risk for bullying victimization (Young et al., 2012). An analysis of data from US Public Schools suggests that disabled youth are 32% more likely to experience bullying victimization than their nondisabled peers (Gage et al., 2021). The present research examines the association of bullying victimization among autistic youth and youth with attention deficit hyperactivity disorder (ADHD) and negative mental health outcomes of anxiety and depression in the US.

## Bullying, Autism and ADHD

Meta-analyses report prevalences of bullying victimization among autistic youth and youth with ADHD in the US and internationally at an alarming 40–60% (e.g., Maiano et al., 2016; Park et al., 2020; Trundle et al., 2023). A meta-analysis by Park et al. (2020) reported the general bullying victimization of autistic youth at 67%, including 58% verbal bullying (e.g., vocal or written speech intended to harm or belittle others), 36% relational bullying (e.g., actions that aim to manipulate, exclude, or isolate the victim), 30% physical bullying (e.g., direct physical harm) and 15% cyber bullying (e.g., bullying that occurs over digital and online platforms). Similarly, Maiano et al. (2016), conducted a meta-analysis and reported the general school bullying victimization of autistic youth at 44%, including 50% verbal bullying, 33% physical, and 31% relational bullying.

In terms of ADHD, Bustinza et al. (2022) conducted a secondary analysis of National Survey of Children’s Health (NSCH) 2016–2017 data and factors associated with bullying and reported 47% of youth with ADHD to be victims of bullying compared to 23% of youth without ADHD. Factors associated with increased bullying victimization were reported to include developmental delay, family need, and difficulty with social peer relationships (Bustinza et al., 2022). Reporting prevalence even higher, Fogler et al. (2022) conducted a secondary analysis of school district data and reported bullying victimization of youth with ADHD at 60%.

## Co-Occurrence of Autism and ADHD and Bullying Victimization

Rates of bullying victimization are reported even higher for youth with co-occurring autism and ADHD diagnoses. The co-occurrence of autism and ADHD is well established, with meta-analyses of international data and nationally representative U.S. datasets commonly reporting 40–50% of autistic youth meeting the criteria for an ADHD diagnosis, and 10–21% of youth with ADHD meeting the criteria for an autism diagnosis (e.g. see Accardo et al., 2022; Hollingdale et al., 2020; Rong et al., 2021; Zablotsky et al., 2020). Researchers report factors increasing the bullying victimization risk of autistic youth to include co-occurring ADHD (Sterzing et al., 2012; Zablotsky et al., 2013), as well as differences in social and communication skills (Matthias et al., 2021; Park et al., 2020; Sterzing et al., 2012), and time spent in general education settings (Park et al., 2020; Sterzing et al., 2012).

While autism and ADHD are increasingly recognized from a strength-perspective with students valued for their neurodivergence in higher education (Dwyer et al., 2023; Nyhan, 2018), within traditional K-12 school systems differences associated with neurodiversity remain surrounded by stigma (e.g. see Earnshaw et al., 2018). Earnshaw et al. (2018) report that youth with characteristics perceived by their peers to not be socially desirable face heightened incidences of bias, stereotypes, stigma-based bullying and social dominance. Similarly, Adams et al. (2016) report autistic students in mainstreamed settings are more likely to be ignored, purposely excluded by their peers, victimized for having intense areas of interest, and/or provoked to exhibit social or emotional responses. Unfortunately, teachers in general and inclusive settings are reported as not equipped to put an end to the bullying of youth with disability labels (Park et al., 2020; Young et al., 2012). Teachers also lack strategies to foster positive relationships between disabled youth and their nondisabled peers (e.g., see Rentenbach et al., 2017), which contributes to the ongoing prevalence of bullying.

## Bullying, Autism, ADHD and Mental Health

Beyond a consideration of bullying prevalence and related factors, there does not appear to be any research that investigates whether the negative effects of bullying on the victim’s mental health are greater among autistic youth and youth with ADHD. This is especially relevant as victimization-related mental health conditions are substantially more prevalent among autistic and ADHD youth (Accardo et al., 2022) and the elevated risk of such mental health conditions may persist for decades (Arseneault, 2017; Arseneault et al., 2010; Bryson et al., 2021; Moore et al., 2017). Autistic youth, youth with ADHD, and youth with co-occurring autism and ADHD experience higher incidences of mental health conditions (Accardo et al., 2022; Hossain et al., 2020; Lai et al., 2019; Zablotsky et al., 2013). For example, a secondary analysis of the multi-year (NSCH 2016–2019 data reports anxiety and depression among autistic youth and/or youth with ADHD aged 12–17 at prevalence rates a troubling ten times their non-autistic, non-ADHD peers (Accardo et al., 2022). Furthermore, a longitudinal study of autistic US youth was conducted by Rodriguez et al. (2021) to examine the impact of bullying victimization on adverse mental health. Results showed that parent reports of their child’s bullying victimization in year one are associated with an increase in the child’s teacher-reported mental health problems in year two, but not vice versa. These results indicate bullying victimization may influence future mental health problems among autistic youth.

Moreover, in a systematic review of 13 articles, Simmons and Antshel (2020) report positive associations among ADHD, bullying, and depression, with depression increasing as a result of bullying. The initial research highlighting this relationship has led scholars to call for future studies to better understand the associations among bullying, ADHD and mental health (Simmons & Antshel, 2020; Rodriguez et al., 2021). Of utmost concern, autistic youth with and without co-occurring ADHD increasingly experience exacerbated mental health conditions, including anxiety and depression, as a result of misunderstanding and stigma-based bullying from non-autistic/ non-ADHD peers (Lebowitz, 2016; Williams et al., 2019).

## Bullying and Sex Differences

In addition to studying the link between bullying victimization and mental health for autistic youth and youth with ADHD, it is vital to explore how these relationships might vary by sex. There is a large variation in diagnostic rates by sex, with males four times as likely to receive an autism diagnosis (Maenner et al., 2023) and twice as likely to receive an ADHD diagnosis (Bitsko et al., 2022) than females. Sex disparities have also been observed concerning bullying experiences, with U.S. school age females reported to experience significantly greater bullying victimization than males (Pontes et al., 2018a, 2022), and with females reporting heightened rates of relational bullying and males reporting heightened rates of direct bullying (Wang et al., 2009). Finally, female victims of bullying report more cyberbullying than males and higher levels of suicidal ideation (Pontes et al., 2018b; Strohacker et al., 2021).

## Aim

Associations among bullying and autism (Maiano et al., 2016; Park et al., 2020; Trundle et al., 2023) as well as bullying and ADHD (Bustinza et al., 2022; Fogler et al., 2022) are commonly reported, however there is a lack of research using large data sets to investigate these associations and limited research exploring the associations between bullying victimization of youth with autism and/or ADHD and mental health conditions (Chou et al., 2020; Iyanda, 2022; Stanyon et al., 2022). The present study investigates whether the association between bullying victimization and diagnosed anxiety or depression among US youth 12–17 years is significantly greater among autistic youth, or non-autistic youth with ADHD, relative to non-autistic non-ADHD youth using data from NSCH. We restricted analyses to youth aged 12–17 years as this population has been reported by the CDC to experience anxiety and depression at heightened rates (CDC, 2022). The major aims of this study are: (1) to examine whether autistic youth or ADHD youth are at greater risk of bullying victimization than their non-autistic non-ADHD peers, and 2) to examine whether bullying victimization is associated with greater increase in the prevalence of anxiety or depression among autistic youth or ADHD youth than among non-autistic non-ADHD youth. We perform analyses separately among male and female youth. To our knowledge this is the first study using multi-year national data sets to investigate whether bullying victimization has larger associations with anxiety and depression in autistic youth, or non-autistic ADHD youth than in non-autistic non-ADHD youth. High rates of bullying victimization among autistic or ADHD youth and large associations between bullying victimization and anxiety or depression among these groups would suggest that the prevention of bullying victimization is important to reduce the high burden of anxiety and depression in these youth. The other major aims of this research are 3) to examine whether the association between an autism and bullying victimization varies by ADHD diagnoses, and 4) to examine whether the magnitude of the association between bullying victimization and anxiety or depression among autistic youth varies by whether or not they have ADHD.

## Rationale for Estimation of Average Marginal Percentages and Additive Interactions

Average marginal percentages should be reported when researchers analyze data with binary dependent variables (Bieler et al., 2010; Leeper et al., 2018; Lumley, 2020; Norton et al., 2019). Consequently, we report average marginal percentages (adjusted for covariates); also referred to as adjusted percentages. STROBE (Strengthening the Reporting of Observational Studies in Epidemiology) guidelines recommend that researchers report additive interactions for binary dependent variables (Vandenbroucke et al., 2007). An additive interaction between autism and bullying victimization on anxiety tests whether the increase in anxiety (increase in the percentage of youth with diagnosed anxiety) associated with bullying victimization does not vary significantly if the youth are autistic or are non-autistic, non-ADHD). Thus, if RD_Au_ is the risk (percentage) difference for the effect of bullying victimization on anxiety among autistic youth and RD_NoAuNoADHD_ is the risk (percentage) difference for the effect of bullying victimization on anxiety among non-autistic non-ADHD youth, then, the null hypothesis is no additive interaction, equivalently the difference, RD_Au_- RD_NoAuNoADHD_ = 0 (Rothman, 2014; Rothman et al., 1980). A significant additive interaction implies that the 95% CI for the difference RD_Au_- RD_NoAuNoADHD_ does not include 0. This research follows STROBE guidelines and reports risk differences and additive interactions to test whether the association between bullying victimization and anxiety is significantly greater among autistic youth (non-autistic ADHD youth) than among non-autistic non-ADHD youth.

## Methods

### Dataset

For the analysis, the research team used the NSCH data. This NSCH data is appropriate for the proposed analysis as it includes data for children, aged zero to 17 years of age, covering their physical and mental health, disabilities, and experiences in bullying victimization. The survey is sponsored by the Maternal and Child Health Bureau of the Health Resources and Services Administration (HRSA), with data housed on a Children and Adolescent Health Measurement Initiative (CAHMI) website (2023). The Census Bureau administers the NSCH annually across the United States by randomly sampling households via internet, telephone, and traditional mail (US Census Bureau, 2024a). According to the HRSA, the survey intentionally oversamples children with disability labels and children ages zero to five to improve the precision of estimates for bullying victimization, depression, and anxiety. The NSCH datasets incorporate essential sampling design variables and sample weights, which need to be incorporated to ensure accurate estimation of population statistics. Previous research has already reported this prevalence (Accardo, et al., 2022) using four years of data, from 2016 to 2019. The present study included five years from 2016 to 2020. The prevalence data overlaps four years out of the five used for this study, and likely has similar results in the fifth year to the previous four. Data were analyzed as recommended using R and the R survey package which incorporates the sampling weights in the analysis. Additional details regarding NSCH’s sampling methodology can be found on the CAHMI website (CAHMI, 2023; US Census Bureau, 2024b).

The team downloaded data from the CAHMI website and prepared a concatenated data set consisting of five years of data (e.g., 2016–2020). The concatenated data set represented 174,551 respondents. Respondents with missing data were excluded from analyses: Autism = 0.53%, ADHD = 1.09%, Anxiety = 0.86%, and Depression = 0.59%. After restricting the results to youth ages 12–17 years (both inclusive years), and to respondents that also had a SCH-T3 survey completed by adults who reside in their same household, the research team obtained a total sample of 71,973 respondents.

### Study Variables

#### Autism-ADHD

The first variable of interest was the autism and ADHD diagnostic status of the youth. The NSCH SCH T3 survey includes a question “Has a doctor or other health care provider EVER told you that this child has Autism or Autism Spectrum Disorder (autism)? *Include diagnoses of Asperger’s Disorder or Pervasive Development Disorder (PDD).*” Possible answers to this question were “yes” or “no”, with “yes” indicating the youth was diagnosed with autism or related spectrum disorder and “no” indicating they had not been diagnosed. Similarly, the SCH T3 survey asks, “Has a doctor or other health care provider EVER told you that this child has Attention Deficit Disorder or Attention Deficit/Hyperactivity Disorder, that is ADD or ADHD?” Like the autism diagnosis question, the possible answers to this question were “yes” or “no”, with “yes” indicating the child was diagnosed with autism or related spectrum disorder and “no” indicating they had not been diagnosed. Analysis of the 71,973 respondents identified a majority of the children were non-autistic non-ADHD youth (n = 59,769), the second most prevalent group were non-autistic ADHD youth (n = 8,948), with a remaining 2,489 youth reporting an autism diagnosis.

#### Bullying Victimization

The next variable of interest was the degree to which the survey respondents reported bullying victimization. For bullying victimization, we used the variable npm9bullied which was available in the dataset downloaded from the Children and Adolescent Health Measurement Initiative (CAHMI) at Johns Hopkins University. For the 2016 and 2017 survey, the survey asked respondents to select either (a) “Definitely true,” (b) “Somewhat true,” or (c) “Not true” in response to the statement “This child is bullied, picked on, or excluded by other children.” CAHMI collapsed the three responses into a binary variable (npm9_bullied) with “Yes” including the responses “Definitely true” and “Somewhat true.” and “No” included the response “Not True.”

The 2018, 2019, and 2020 survey, the question varied a bit from the 2016 and 2017 survey. Survey respondents were asked to respond to the statement “DURING THE PAST 12 MONTHS, how often was this child bullied, picked on, or excluded by other children? If the frequency changed throughout the year, report the highest frequency.” For this statement, survey respondents were asked to select either (a) “Never,” (b) “1–2 times (in the past 12 months),” (c) “1–2 times per month,” (d) “1–2 times per week, or (e) “Almost every day.” CAHMI collapsed the responses into a binary variable (npm9_bullied) with “Yes” including any of the positive responses (options b-e) and “No” included the response “Never.” Note: the variable nmp9_bullied has been used to estimate National Performance Measure 9; the prevalence of bullying victimization among US youth 12–17 years.

#### Anxiety

The next variable of interest was the degree to which the survey respondents reported anxiety. The NSCH SCH T3 survey includes a question “Has a doctor or other health care provider EVER told you that this child has Anxiety Problems?” Possible answers to this question were “yes” or “no,” with “yes” indicating the child was diagnosed with anxiety and “no” indicating they had not been diagnosed.” The survey also included a question “If yes, does this child CURRENTLY have the condition?” Possible answers to this question were “yes” or “no.” To prepare the data set, if the respondents answered “Yes” to both questions, they were coded as “Yes.” If they responded “No” to either question, they were coded as “No.”

#### Depression

The next variable of interest was the degree to which the survey respondents reported depression. The NSCH SCH T3 survey includes a question “Has a doctor or other health care provider EVER told you that this child has Depression?” Possible answers to this question were “yes” or “no,” with “yes” indicating the child was diagnosed with depression and “no” indicating they had not been diagnosed. The survey also included a question “If yes, does this child CURRENTLY have the condition?” Possible answers to this question were “yes” or “no.” Similar to the anxiety variable, to prepare the data set, if the respondents answered “Yes” to both questions, they were coded as “Yes.” If they responded “No” to either question, they were coded as “No.”

#### Anxiety and Depression

Using the variables of anxiety and depression, the research team stratified the youth into two groups: (1) “yes” identifying the youth had been diagnosed with both anxiety and depression or (2) “no” identifying all other youth. Missing data were coded as missing.

#### Demographic Data

The demographic variables for this analysis included sex (male or female), age (in years), year of survey, and race/ethnicity (non-Hispanic White, non-Hispanic Black, Hispanic, and non-Hispanic Other). To prepare the data for analysis, the research team transformed the variables of “autism-ADHD,” “anxiety,” “depression,” “anxiety and depression,” “sex,” “race/ethnicity,” “year of survey,” and “age” into categorical variables.

### Analyses

For this research, we prepared an analytic dataset by combining five years of data (2016–2020) from the NSCH; a multi-year dataset has a greater sample size that increases the power of a study (CAHMI, 2023; US Census Bureau, 2024a, 2024b). The greater sample size (increased power) enables more reliable estimates for the association between bullying victimization and anxiety or depression among smaller population groups (US Census Bureau, 2024a), such as female autistic youth who are less commonly diagnosed compared to males (Accardo et al., 2022). The increased power from a multi-year study is also needed to estimate interactive effects between bullying victimization x neurodiversity since interactive effects typically require much larger sample sizes than the samples used to test for the main effects (Gelman, 2018). Of note, to address the extensive overlap between autism and ADHD, additional analyses were conducted to estimate whether autistic youth with co-occurring ADHD are at an especially high risk for bullying victimization, anxiety or depression, and whether the association between bullying victimization and anxiety or depression was significantly greater among those with both autism and ADHD.

The open-sourced R software and the R survey package were used to perform multivariate analyses and generate nationally representative weighted estimates (Lumley, 2020; R Core Team, 2020). The R survey package function, “svypredmeans,” was used to estimate average marginal percentages, and the R survey package function, “svycontrast,” was used to estimate adjusted risk differences, additive interactions, and their respective confidence intervals from marginal means as described (Bieler et al., 2010; Lumley, 2020).

## Results

### Association Between Autism/ADHD and Bullying Victimization by Sex

**Fig. 1:**
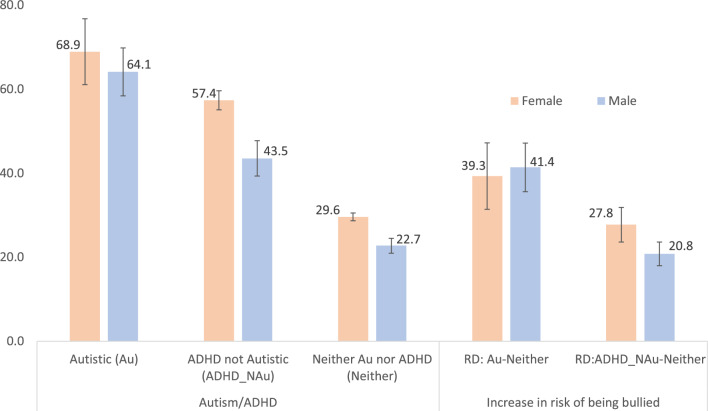
Average Marginal Percentage of US Youth 12–17 years who were Bullied Stratified by Autism/ADHD and Sex (RD = Percentage Increase in Bullying Victimization Associated with Autism/ADHD Stratified by Sex

Among male and female US youth (12–17 years), the prevalence of bullying victimization was highest among autistic female (68.9%) and male (64.1%) youth (Table 2 and Fig. 1). Among female youth, the average marginal percentage of bullying victimization was lowest among those who were non-autistic non-ADHD (29.6%) (Table 2 and Fig. 1). Comparatively, bullying victimization was significantly greater among autistic female youth (68.9%, risk [percentage] difference [RD] = 39.3%, 95% CI = [31.4, 47.2],* t* = 9.44, *p* < 0.001) and non-autistic ADHD female youth (57.4%, RD = 27.7%, 95% CI = [23.6, 31.9], *t* = 13.21, *p* < 0.001) (Tables 1 and 2 and Fig. 1).

**Table 1:** Average marginal percentage of US youth 12–17 years who were bullied stratified by autism/ADHD and sex

Sex	% (SE) of Youth (12–17 years) who were bullied		
	Autistic	Non-Autistic ADHD	Non-Autistic non-ADHD (Ref)
Female	68.9 (4.00)	57.4 (2.04)	29.6 (0.58)
Male	64.1 (2.91)	43.5 (1.35)	22.7 (0.52)

*%* percentage of US youth who were victims of bullying, *SE* standard error of estimate

**Table 2:** Percentage increase in the prevalence of bullying victimization associated with autism/ADHD stratified by sex (Ref = non-autistic non-ADHD youth)

Sex	Autistic			ADHD non-Autistic		
	RD [95% CI]	*t*	*p*	RD [95% CI]	*t*	*p*
Female	39.3 [31.4, 47.2]	9.74	< .001	27.7 [23.6, 31.9]	13.21	< .001
Male	41.4 [35.6, 47.2]	14.01	< .001	20.8 [ 18.0, 23.6]	14.49	< .001

*RD* risk (percentage) difference, *95% CI* 95% confidence interval, *t* *t* statistic, *p* probability (significance level)

Similarly, among male youth 12–17 years, the average marginal percentage of bullying victimization was lowest among those who were non-autistic non-ADHD (22.7%) (Table 2 and Fig. 1). Bullying victimization was also significantly greater among autistic male youth (64.1%, RD = 41.4%, 95% CI = [35.6, 47.2], *t* = 14.01, *p* < 0.001, and non-autistic ADHD male youth (43.5%, RD = 20.8%, 95% CI = [18.0, 23.6], *t* = 14.49, *p* < 0.001 (Table 2 and Fig. 1).

### Association Between Bullying Victimization and Anxiety is Greater Among Autistic and ADHD Youth

**Fig. 2:**
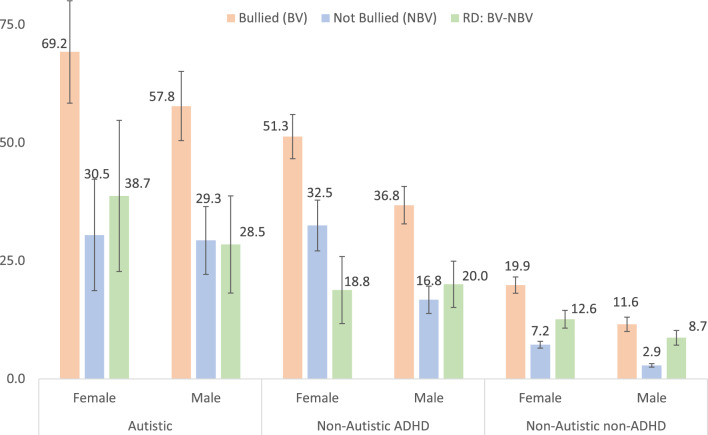
Average Marginal Percentage of Anxiety in US Youth 12–17 years Stratified by Autism/ADHD, Sex, and Bullying Victimization (RD = Percentage Increase in Anxiety Associated with Bullying Victimization Stratified by Autism/ADHD and Sex)

There were significant positive associations between bullying victimization and anxiety among autistic youth, non-autistic ADHD youth, or non-autistic non-ADHD youth. Among female youth, the average marginal percentage of anxiety was significantly greater among those who experienced bullying victimization than among those who experienced no bullying victimization (Autistic youth: 69% versus 30.5%, RD = 38.7%, *t* = 4.75, *p* < 0.001; non-Autistic ADHD youth: 51.3% versus 32.5%, RD = 18.8%, *t* = 5.21, *p* < 0.001; non-Autistic non-ADHD youth: 19.9% versus 7.2%, RD = 12.6%,* t* = 13.11, *p* < 0.001 (Table 4 and Fig. 2). Additive interactions show that the increase in anxiety associated with bullying victimization among female youth was significantly greater among autistic youth (38.7%) than among non-autistic non-ADHD youth, RD = 12.6%, AI_RD = 26.1%, *t* = 3.18, *p* = 0.001 (Table 4). The increase in anxiety associated with bullying victimization was not significantly greater among non-autistic ADHD youth (18.8%) than among non-autistic non-ADHD youth, RD = 12.6%, AI_RD = 6.2%, *t* = 1.66, *p* = 0.097 (Table 4).

Among male youth, the average marginal percentage of anxiety was significantly greater among male youth who experienced bullying victimization than among those who experienced no bullying victimization (Autistic males: 57.8% versus 29.3%, RD = 28.5%, *t* = 5.43, *p* < 0.001; non-Autistic ADHD males: 36.8% versus 16.8%, RD = 20.0%, *t* = 8.01, *p* < 0.001; non-Autistic non-ADHD youth: 11.6% versus 2.9%, RD = 8.7%, *t* = 10.96, *p* < 0.001 (Tables 3 and 4 and Fig. 2). Additive interactions show that the increase in anxiety associated with bullying victimization among male youth was significantly greater among autistic youth (28.5%) than among non-autistic non-ADHD youth, RD = 8.7%, AI_RD = 19.8%,* t* = 3.73, *p* < 0.001 (Table 4). The increase in anxiety associated with bullying victimization was also significantly greater among non-autistic ADHD youth (18.8%) than among non-autistic non-ADHD youth, RD = 8.7%, AI_RD = 11.3%, *t* = 4.34, *p* < 0.001 (Table 4).

**Table 3:** Average marginal percentage of US youth 12–17 years with anxiety stratified by autism/ADHD and bullying victimization (BV)

Sex	% (SE) of Youth (12–17 years) who had diagnosed anxiety					
	Autistic (Au)		Non-autistic ADHD		Non-autistic non-ADHD (Ref)	
	BV	No BV	BV	No BV	BV	No BV
Female	69.2 (5.53)	30.5 (6.02)	51.3 (2.39)	32.5 (2.74)	19.9 (0.88)	7.2 (0.37)
Male	57.8 (3.73)	29.3 (3.66)	36.8 (2.02)	16.8 (1.47)	11.6 (0.77)	2.9 (0.19)

*%* percentage of US youth who were diagnosed with anxiety, *SE* standard error of estimate

**Table 4:** Percentage increase in the prevalence of anxiety among US youth 12–17 years associated with bullying victimization stratified by autism/ADHD and sex

Autism/ADHD	Sex	RD [95% CI]	*t*	*p*	AI_RD [95% CI]	*t*	*p*
Autistic	Female	38.7 [22.7, 54.7]	4.75	< .001	26.1 [10.0, 42.2]	3.18	.001
	Male	28.5 [18.2, 38.7]	5.43	< .001	19.8 [9.4, 30.1]	3.73	< .001
Non-Autistic	Female	18.8 [11.7, 25.9]	5.21	< .001	6.2 [-1.1, 13.5]	1.66	.097
ADHD	Male	20.0 [15.1, 24.9]	8.01	< .001	11.3 [6.2, 16.4]	4.34	< .001
Non-Autistic	Female (Ref)	12.6 [10.7, 14.5]	13.11	< .001			
non-ADHD	Male (Ref)	8.7 [7.2, 10.3]	10.96	< .001			

*RD* risk (percentage) increase in the prevalence of anxiety associated with bullying victimization, *95% CI* 95% confidence interval, *t* *t* statistic, *p* probability (significance level), *AI_RD* additive interaction (increase in anxiety associated with bullying victimization is greater in one group versus the control group)

### Association Between Bullying Victimization and Depression is Greater Among Autistic/ADHD Youth

**Fig. 3:**
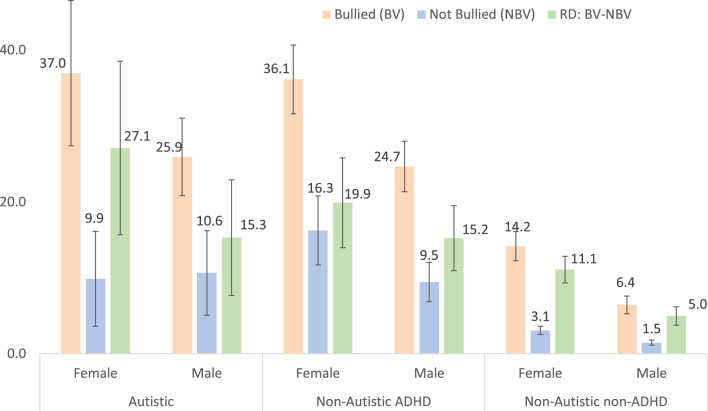
Average Marginal Percentage of Depression in US Youth 12–17 years Stratified by Autism/ADHD, Sex, and Bullying Victimization (RD = Percentage Increase in Depression Associated with Bullying Victimization Stratified by Autism/ADHD and Sex)

Results show significant positive associations between bullying victimization and depression among autistic youth, non-autistic ADHD youth, and non-autistic non-ADHD youth. Among female youth 12–17 years, the average marginal percentage of depression was significantly greater among those who experienced bullying victimization than among those who experienced no bullying victimization (Autistic youth: 37.0% versus 9.9%, RD = 27.1%, *t* = 4.65, *p* < 0.001; non-Autistic ADHD youth: 36.1% versus 16.3%, RD = 19.9%, *t* = 6.59, *p* < 0.001; non-Autistic non-ADHD youth: 14.2% versus 3.1%, RD = 11.1%, *t* = 12.41, *p* < 0.001 (Table 6 and Fig. 3). Additive interactions show that the increase in depression associated with bullying victimization among female youth was significantly greater among those who were autistic (RD = 27.1%) than among those who were non-autistic non-ADHD (RD = 11.1%), AI_RD = 16.0%, *t* = 2.72, *p* = 0.007 (Table 6). The increase in depression associated with bullying victimization was also significantly greater among non-autistic ADHD youth (RD = 19.9%) than among non-autistic non-ADHD youth (RD = 11.1%), AI_RD = 8.8%,* t* = 2.82, *p* = 0.005 (Table 6).

Among male youth 12–17 years, the average marginal percentage of depression was significantly greater among those who experienced bullying victimization than among those who experienced no bullying victimization (Autistic youth: 25.9% versus 10.6%, RD = 15.3%,* t* = 3.94, p < 0.001; non-Autistic ADHD youth: 24.7% versus 9.5%, RD = 15.2%, *t* = 6.98, *p* < 0.001; non-Autistic non-ADHD youth: 6.4% versus 1.5%, RD = 5.0%,* t* = 7.98, *p* < 0.001 (Tables 5 and 6 and Fig. 3). Additive interactions show that the increase in depression associated with bullying victimization among male youth was significantly greater among those who were autistic (RD = 15.3%) than among those who were non-autistic non-ADHD (RD = 5.0%), AI_RD = 10.3%,* t* = 2.63, *p* = 0.009. The increase in depression associated with bullying victimization was also significantly greater among non-autistic ADHD youth (15.2%) than among non-autistic non-ADHD youth (RD = 5.0%), AI_RD = 10.3%, *t* = 4.58, *p* < 0.001.

**Table 5:** Average marginal percentage of US youth 12–17 years with diagnosed depression stratified by autism/ADHD, sex, and bullying victimization (BV)

Sex	% (SE) of youth (12–17 years) who had diagnosed depression					
	Autistic		Non-autistic ADHD		Non-autistic non-ADHD (Ref)	
	BV	No BV	BV	No BV	BV	No BV
Female	37.0 (4.88)	9.9 (3.19)	36.1 (2.28)	16.3 (2.02)	14.2 (0.86)	3.1 (0.22)
Male	25.9 (2.61)	10.6 (2.85)	24.7 (1.70)	9.5 (1.33)	6.4 (0.60)	1.5 (0.17)

*%* percentage of US youth who were diagnosed with depression, *SE* standard error of estimate

**Table 6:** Percentage increase in the prevalence of depression among US youth 12–17 years associated with bullying victimization stratified by autism/ADHD and sex

Neurodiversity	Sex	RD [95% CI]	*t*	*p*	AI_RD [95% CI]	*t*	*p*
Autistic	Female	27.1 [15.7, 38.5]	4.65	< .001	16.0 [4.5, 27.6]	2.72	.007
	Male	15.3 [7.7, 22.9]	3.94	< .001	10.3 [2.6, 18.0]	2.63	.009
Non-Autistic	Female	19.9 [14.0, 25.8]	6.59	< .001	8.8 [2.7, 14.9]	2.82	.005
ADHD	Male	15.2 [11.0, 19.5]	6.98	< .001	10.3 [5.9, 14.7]	4.58	< .001
Non-Autistic	Female	11.1 [9.3, 12.8]	12.41	< .001			
non-ADHD	Male	5.0 [3.7, 6.2]	7.98	< .001			

*RD* risk (percentage) increase in the prevalence of depression associated with bullying victimization, *95% CI* 95% confidence interval, *t* *t* statistic, *p* probability (significance level), *AI_RD* additive interaction (increase in depression associated with bullying victimization is greater in one group versus the control group)

### Association Between Bullying Victimization and Depression with Anxiety is Greater Among Autistic/ADHD Youth

**Fig. 4:**
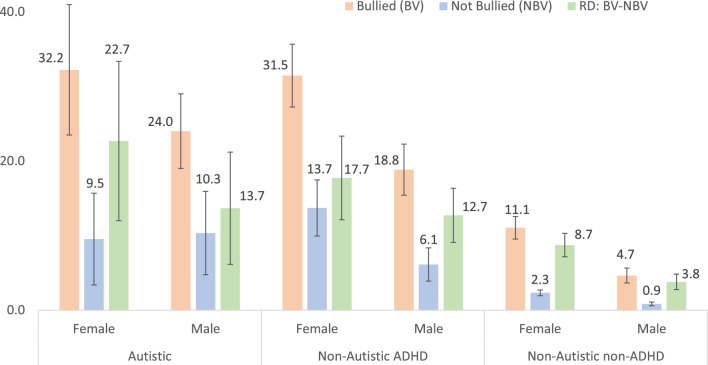
Average Marginal Percentage of Anxiety with Depression in US Youth 12–17 years Stratified by Autism/ADHD, Sex, and Bullying Victimization (RD = Percentage Increase Anxiety with Depression Associated with Bullying Victimization Stratified by Autism/ADHD and Sex

Results show significant positive associations between bullying victimization and depression among autistic youth, non-autistic ADHD youth, and non-autistic non-ADHD youth. Among female youth, the average marginal percentage of depression with anxiety was significantly greater among those who experienced bullying victimization than among those who experienced no bullying victimization (Autistic youth: 32.2% versus 9.5%, RD = 22.7%, *t* = 4.16, *p* < 0.001; non-Autistic ADHD youth: 31.5% versus 13.7%, RD = 17.7%, *t* = 6.21, *p* < 0.001; non-Autistic non-ADHD youth: 11.1% versus 2.3%, RD = 8.7%, *t* = 10.89, *p* < 0.001 (Tables 7 and 8 and Fig. 4). Additive interactions show that the increase in depression with anxiety associated with bullying victimization among female youth was significantly greater among those who were autistic (RD = 22.7%) than among those who were non-autistic non-ADHD (RD = 8.7%), AI_RD = 13.9%, *t* = 2.53, *p* = 0.011. The increase in depression with anxiety associated with bullying victimization was also significantly greater among non-autistic ADHD youth (RD = 17.7%) than among non-autistic non-ADHD youth (RD = 8.7%), AI_RD = 9.0%, *t* = 3.06, *p* = 0.002.

**Table 7:** Prevalence of anxiety with depression among US youth 12–17 years stratified by autism/ADHD and bullying victimization (BV)

Sex	% (SE) of youth (12–17 years) who had anxiety with depression					
	Autistic (Au)		Non-autistic ADHD		Non-autistic non-ADHD (Ref)	
	BV	No BV	BV	No BV	BV	No BV
Female	32.2 (4.46)	9.5 (3.14)	31.5 (2.15)	13.7 (1.92)	11.1 (0.78)	2.3 (0.19)
Male	24.0 (2.55)	10.3 (2.83)	18.8 (1.49)	6.1 (1.08)	4.7 (0.51)	0.9 (0.13)

*%* percentage of US youth who were diagnosed with both anxiety and depression, *SE* standard error of estimate

**Table 8:** Increase in the prevalence of anxiety with depression among US youth 12–17 years associated with bullying victimization stratified by neurodiversity and sex

Neurodiversity	Sex	RD [95% CI]	*t*	*p*	AI_RD [95% CI]	*t*	*p*
Autistic	Female	22.7 [12.0, 33.4]	4.16	< .001	13.9 [3.1, 24.7]	2.53	.011
	Male	13.7 [6.1, 21.2]	3.56	< .001	9.9 [2.3, 17.4]	2.55	.011
Non-Autistic	Female	17.7 [12.1, 23.3)	6.21	< .001	9.0 [3.2, 14.8]	3.06	.002
ADHD	Male	12.7 [9.1, 16.4]	6.86	< .001	8.9 [5.2, 12.6]	4.68	< .001
Non-Autistic	Female	8.7 [7.2, 10.3]	10.89	< .001			
non-ADHD	Male	3.8 [2.8, 4.8]	7.24	< .001			

*RD* risk (percentage) increase in the prevalence of depression associated with bullying victimization, *95% CI* 95% confidence interval, *t* *t* statistic, *p* probability (significance level), *AI_RD* additive interaction (increase in depression associated with bullying victimization is greater in one group versus the control group)

Among male youth 12–17 years, the average marginal percentage of depression with anxiety was significantly greater among those who experienced bullying victimization than among those who experienced no bullying victimization (Autistic youth: 24.0% versus 10.3%, RD = 13.7%, *t* = 3.56, *p* < 0.001; non-Autistic ADHD youth: 18.8% versus 6.1%, RD = 12.7%,* t* = 6.86, *p* < 0.001; non-Autistic non-ADHD youth: 4.7% versus 0.9%, RD = 3.8%, *t* = 7.24, *p* < 0.001 (Tables 7 and 8 and Fig. 4). Additive interactions show that the increase in depression with anxiety associated with bullying victimization among male youth was significantly greater among those who were autistic (13.7%) than among those who were non-autistic non-ADHD (RD = 3.8%), AI_RD = 9.9%, *t* = 2.55, *p* = 0.011. The increase in depression with anxiety associated with bullying victimization was also significantly greater among non-autistic ADHD male youth (RD = 12.7%) than among non-autistic non-ADHD male youth (RD = 3.8%), AI_RD = 8.9%, *t* = 4.68, *p* < 0.001.

### Association Between Autism and Bullying Victimization by ADHD

Results show the prevalence of bullying victimization was highest among youth with co-occurring autism and ADHD (71.7%) (Table 9). Among youth with ADHD, the prevalence of bullying victimization was significantly greater among autistic youth (71.7%) than among non-autistic youth (49.5%), RD = 22.2%,* t* = 8.22, *p* < 0.001. Similarly, among youth without ADHD, the prevalence of bullying victimization was significantly greater among autistic youth (63.0%) than among non-autistic youth (26.1%), RD = 36.9%, *t* = 8.75, *p* < 0.001.

**Table 9:** Average marginal percentage of US youth 12–17 years who were bullied stratified by autism and ADHD

ADHD	% (SE) of Youth (12–17 years) who were bullied				
	Autistic	Non-Autistic (Ref)	RD [95% CI]	*t*	*p*
ADHD	71.7 (2.48)	49.5 (1.15)	22.2 [16.9, 27.5]	8.22	< .001
No ADHD	63.0 (4.20)	26.1 (0.39)	36.9 [28.6, 45.1]	8.75	< .001

*%* percentage of US youth who were victims of bullying, *SE* standard error of estimate

### Association Between Bullying Victimization and Anxiety Among Autistic Youth by ADHD

Results show that among autistic youth, the prevalence of anxiety was highest among those with co-occurring ADHD who were bullied (75.0%) (Tables 9, 10). Among autistic youth with co-occurring ADHD, the prevalence of anxiety was significantly greater among bullied youth (75.0%) than among youth who were not bullied (50.0%), RD = 25.0%, t = 4.32, p < 0.001. Similarly, among autistic youth without co-occurring ADHD, the prevalence of anxiety was significantly greater among bullied youth (52.2%) than youth who were not bullied (22.2%), RD = 36.9%, t = 8.75, p < 0.001.

**Table 10:** Association between bullying victimization and anxiety, depression among autistic youth by ADHD

Diagnosed	ADHD	Bullied—% (SE)		RD [95% CI]	*t*	*p*
		Yes	No			
Anxiety	Yes	75.0 (2.80)	50.0 (5.12)	25.0 [13.6, 36.3]	4.32	< .001
	No	52.2 (6.04)	22.2 (3.74)	30.0 [16.1, 43.9]	4.22	< .001
Depression	Yes	41.8 (3.23)	22.5 (5.37)	19.3 [7.0, 31.5]	3.08	.002
	No	19.2 (3.14)	4.8 (2.05)	14.4 [7.1, 21.8]	3.85	< .001

*RD* risk (percentage) increase in the prevalence of anxiety (depression) associated with bullying victimization, *95% CI* 95% confidence interval, *t* t statistic, *p* probability (significance level)

### Association Between Bullying Victimization and Depression Among Autistic Youth by ADHD

Results show that among autistic youth, the prevalence of depression was highest among those with co-occurring ADHD who were bullied (41.8%) (Table 10). Among autistic youth with co-occurring ADHD, the prevalence of depression was significantly greater among bullied youth (41.8%) than among youth who were not bullied (22.5%), RD = 19.3%, *t* = 3.08, *p* = 0.002). Among autistic youth without ADHD, the prevalence of depression was also significantly greater among bullied youth (19.2%) than among youth who were not bullied (4.8%), RD = 14.4%, *t* = 3.85, *p* < 0.001. Among autistic youth without ADHD who were not bullied, the relative standard error is greater than 30% (the standard error of the estimate is more than 30% of the estimate), therefore, as per guidelines, this estimate is unreliable and should either not be reported or flagged (Health Resources & Services Administration, 2023).

## Discussion

The present study aimed to address a gap in the research relative to school age autistic youth, youth with ADHD, youth with co-occurring autism and ADHD, bullying victimization, and associations with the mental health conditions of anxiety and depression (Chou et al., 2020; Iyanda, 2022; Stanyon et al., 2022). To our knowledge this is the first study to use multi-year, national data to identify: (1) prevalence of bullying victimization of autistic youth, youth with ADHD, and non-autistic/non-ADHD youth stratified by sex, (2) prevalence of anxiety among autistic youth, youth with ADHD, and non-autistic/non-ADHD youth experiencing bullying victimization stratified by sex, and (3) prevalence of depression among autistic youth, youth with ADHD, and non-autistic/non-ADHD youth experiencing bullying victimization stratified by sex.

### Key Findings

#### Associations Between Bullying Victimization and Anxiety, Depression are Significantly Greater Among Autistic and ADHD Youth

The major contribution of this study is the use of nationally representative data from the National Survey of Children’s Health to show that among US youth, 12–17 years, the increase in anxiety or depression associated with bullying victimization is significantly and substantially greater among autistic or ADHD youth than among non-autistic youth without ADHD. Among female youth, the increase in anxiety associated with bullying victimization is 3.1 × greater among autistic female youth (38.7%) and 1.5 × greater among non-autistic ADHD female youth (18.8%) relative to non-autistic female youth with no ADHD (12.6%). Similarly, among male youth, the in anxiety associated with bullying victimization is 3.3 × greater among autistic male youth (28.5%) and 2.3 × greater among non-autistic ADHD male youth (20.0%) relative to non-autistic male youth with no ADHD (8.7%).

Similar results are found for the associating of bullying victimization with depression. Among female youth, the increase in depression associated with bullying victimization is 2.4 × greater among autistic female youth (27.1%) and 1.8 × greater among non-autistic ADHD female youth (19.1%) relative to non-autistic female youth with no ADHD (11.1%). Similarly, among male youth, the increase in depression associated with bullying victimization is 3.1 × greater among autistic male youth (15.3% and 3.0 × greater among non-autistic ADHD male youth (15.2%) relative to non-autistic male youth with no ADHD (5.0%).

Reijntjes et al. (2010) report internalizing problems encompassing mental health conditions to have both causal and consequential relationships with bullying victimization, referring to the loop as a “vicious cycle” (p. 251). Results of the present study strengthen the need to recognize and break this potential loop, as findings suggest the vicious mental health and bullying cycle is significantly exacerbated for autistic and/or ADHD youth.

#### Rates of Bullying Victimization are Elevated for Autistic Youth with ADHD

Results of the present study also show that the rates of bullying victimization are significantly heightened among autistic youth with co-occurring ADHD (72%) followed by autistic youth without ADHD (63%) and non-autistic youth with ADHD (50.0%) compared to non-autistic and non-ADHD youth (26.1%). Notably, autistic children with ADHD had a nearly threefold increase in bullying victimization compared to their non-autistic, non-ADHD peers (72% compared to 26%). The present study provides important findings using a large, multi-year data set to build on and strengthen prior research that autistic children with ADHD are at heightened risk for bullying victimization (Sterzing et al., 2012; Zablotsky et al., 2013).

Specific to autism, previous research by Park et al. (2020) estimated 67% of autistic school age youth are victims of bullying. Findings from the present research align with these findings and further identify variation by sex, with 69% of autistic females and 64% of autistic males experiencing bullying victimization, a significant 40% increase in comparison to non-autistic peers. Specific to ADHD, Bustinza et al. (2022) estimated 47% of children with ADHD are victims of bullying, including autistic youth with ADHD. Findings from the present research align with these findings and further identify variation by sex, with 57.4% of females with ADHD and 43.5% of males with ADHD experiencing bullying victimization, a roughly 25% increase in comparison to non-ADHD peers. Findings that autistic females and females with ADHD are more frequently victims of bullying victimization exemplify the need for screening and supports, specifically in relation to preventing and unpacking relational bullying, experienced at increased rates by females (Wang et al., 2009).

#### Rates of Anxiety and Depression are Elevated for Autistic Youth and Youth with ADHD Experiencing Bullying Victimization

Heightened rates of anxiety and depression have been reported for youth with ADHD and/or autism throughout the research literature (e.g., Hossain et al., 2020; Mitchison et al., 2019). Hossain et al. (2020) note the troubling variability of reported prevalence rates for autistic youth in the research literature ranging from 1.5 to 54% for anxiety and 2.5 to 47% for depression. Results of the present study show an even higher trend in anxiety and/or depression among autistic youth who are victims of bullying with anxiety co-occurring among 69% of females and 58% of males, and depression co-occurring among 27% of females and 26% of males. Similarly, among youth with ADHD, previous research notes the prevalence of anxiety to be 42% and depression to be 21% (Mitchison & Njardvik, 2019) yet results of the present study show an even higher trend in anxiety and/or depression among youth with ADHD who are victims of bullying with anxiety co-occurring among 51% of females and 37% of males, and depression co-occurring among 36% of females and 24% of males. Notably, the prevalence of anxiety *with* depression for victims of bullying was much greater among autistic youth (female, 32%; male, 24%) or non-autistic ADHD youth (female, 32%; male, 19%) than among non-autistic non-ADHD youth (female, 11%; male, 5%). Females reported higher rates of anxiety and depression than males. In the present study, this finding aligns with heightened reporting of bullying victimization among females. Finally, youth with co-occurring autism and ADHD reported the highest rates of anxiety and/or depression. These findings highlight a need to screen for, and recognize separate co-occurring neurodevelopmental (e.g. autism and ADHD) and mental health (e.g. anxiety and depression) conditions to ensure youth receive access to supports relevant to each condition as well as a need to ensure characteristics of mental health conditions are not mistaken as related to autistic and/ or youth with ADHD’s primary diagnoses (e.g., see Hossain et al., 2020).

### Future Research

Findings of bullying victimization at a stark 40% increase for autistic peers and 25% increase for peers with ADHD in comparison to non-autistic/non-ADHD youth, coupled with concerning elevated rates of anxiety and/or depression suggest a need for future cause-and -effect research to determine if bullying victimization is causing anxiety/depression, if anxiety/depression is increasing bullying victimization by peers, or if they are working together to create a sustained and detrimental loop impacting autistic and ADHD school age youth (Reijntjes et al., 2010). Further research is warranted to better understand the findings of increased bullying victimization, anxiety and depression of youth with co-occurring autism and ADHD over autistic youth and youth with ADHD, and of females over males, with the exploration of variables beyond those available in the data set of the present study. Future research should evaluate whether supports for mental health conditions also support a reduction in bullying victimization and likewise, if ending bullying victimization supports a reduction in anxiety/depression for school age with autism and/or ADHD.

Negative social interactions including bullying are reported to not only increase mental health conditions, but to increase youth feelings of negativity around their identity (e.g., see Berkovits et al., 2020). Bullying may also lead to social isolation which may further disrupt the formation of friendships. Future research is recommended to consider if bullying victimization and mental health conditions have a direct relationship with youth formation of social ties as well as with youth’s formation of sense-of-self, including disability identity. Similarly further research is recommended to form screening and support recommendations for counselors and psychologists working with autistic youth and/or youth with ADHD with co-occurring mental health conditions (e.g. research may inform counseling practice to screen for past and present bullying victimization as well as to form recommendations as to how treatment of depression, anxiety and other mental health conditions experienced by autistic youth and/or youth with ADHD should adjust to best support youth victims of bullying).

### Implications for Practice

Prior researchers exploring predictors of bullying victimization among autistic youth identify communication and peer interaction differences (Matthias et al., 2021; Park et al., 2020), and time spent in inclusive settings (Park et al., 2020), as factors leading to higher incidences of bullying victimization. Although the cause of bullying victimization is beyond the scope of the present analyses, prior research reports youth with disability labels, including autism and/or ADHD, are more commonly victims of stigma-based victimization as a result of exhibiting differences not deemed socially desirable by peers (e.g., see Earnshaw et al., 2018). While recommendations to reduce bullying victimization often focus on the victim, newer research supports efforts focused on neurotypical and/or nondisabled peers. Specifically anti-stigma education and intergroup contact initiatives might reduce formation of bias and stigma in neurotypical and/or nondisabled peers (Earnshaw et al., 2018; Sterzing et al., 2012).

Recognizing heightened bullying victimization of school age neurodivergent youth as a result of stigma warrants a shift to initiatives that value and respect student differences, through education and practice. Therefore, practitioners might provide education to neurotypical / non-disabled peers focused on recognizing and reducing the power imbalances and stigma around disability and/ or neurodivergence, and establishing practices that proactively prevent negative peer interactions (e.g., ignoring, provoking, and excluding neurodivergent youth; Adams et al., 2016). In fact, some of these efforts are ongoing. For example, there are relatively new state mandates requiring public schools to teach about the contributions of disabled people (e.g., see New Jersey Department of Education, 2019), diversity, equity and inclusion initiatives that center disability as diversity (e.g., Dwyer et al., 2023), and professional development aimed at fostering strengths/asset-based understandings of neurodiversity for school staff, teachers and administrators (Armstrong, 2012). To advance the field, practitioners and researchers might collaborate to evaluate existing evidence-based bullying prevention programs and assess potentially broadening to include anti-stigma initiatives specific to autism and ADHD labels. The emphasis on stigma reduction and education aligns with Park et al. (2020) call for “intervention protocols” for educators to put an end to bullying victimization as well as Morton et al. (2021) call to better understand the bullying experiences of neurodivergent youth.

We have added to the literature by reporting bullying and mental health associations of autistic youth and youth with ADHD by sex. Our findings suggest females with disability labels of autism and ADHD are at increased risk for bullying and associated mental health conditions. Building on the recommendations of Gage et al. (2021), practitioner anti-bullying initiatives might take an intersectional lens, recognizing and valuing all human variations. The concept of intersectionality (Crenshaw, 1989) aligns with the need to consider how intersecting identities relate to power imbalances for multiply marginalized populations (e.g. recognizing ableism and sexism in connection to heightened bullying and mental health needs of females with autism and/or ADHD labels). This is an important step in taking the onus of bullying prevention off the victim, shifting the focus from victim characteristics to initiatives that value diversity.

The present findings emphasize the need for teachers, school personnel and administrators to prioritize support of neurodivergent students, not only through bullying identification, but also through the use of neurodiversity affirming practices. For example, universal support and communication from teachers, such as not singling out students in the classroom, has been suggested to align with autistic students’ feelings of belonging and impact the development of friendships and overall mental health (Williams et al., 2019). Providing teachers with professional development around neurodiversity affirming actions could be a promising addition to existing bullying prevention programs. Rentenbach et al. (2017) provide clear actions teachers can take that strengthen relationships between neurodivergent students and their neurotypical peers such as respecting nonverbal communication and spotlighting students’ strengths. Curriculum and resources to teach about neurodiversity school-wide are emerging and include the Learning About Neurodiversity at School (LEANS) project which provides free K-12 classroom resources, and includes mental health and anti-bullying tools, to support all students in learning about neurodiversity (Alcorn et al., 2022).

### Limitations

Limitations in the present study include those inherent in secondary data analysis with cross-sectional data. Analyses were limited to the existing NSCH parent self-report data. Self-reporting by parents of factors including bullying victimization, physician confirmed autism and/or ADHD diagnoses and current conditions of autism and/or ADHD may be over or under reported, may have been impacted by bias or misunderstanding, and may not capture the same data that school age youth would self-report. Reporting of bullying may also have been negatively impacted by youth not recognizing they are being bullied (e.g. youth may not recognize being ostracized as bullying victimization and/or this may not be reported by parents). Moreover, bullying victimization by caregivers, and bullying by type (e.g., physical, verbal, relational, cyber) were not explored in the present study. Bullying may have been underrepresented in 2016–17 NSCH data as reporting of bullying in 2016–2017 was indicated by the prompt “is” the child bullied; this shifted to the prompt “during the past 12 months how often was this child bullied” beginning 2018. It is possible that the wording “during the past 12 months” may have led to respondents reporting bullying more often than the present term “is.” Of note, consideration was given to excluding 2016–2017 NSCH data, however a decision was made to include the data as it was in alignment with other annual data and if left out, there were not enough females not bullied for the data to be statistically significant. In-person and cyber bullying victimization may also have been impacted in 2020–2021 NSCH data as many students remained home nationally during the COVID pandemic. The study may not capture school age youth identifying as autistic and/or ADHD and/or experiencing anxiety/depression that have not received a clinical diagnosis for a myriad of reasons including low socioeconomic status, lack of access to health care services, and stigma associated with autism and ADHD labels and mental health conditions. Finally, the survey asks parents the question, "What is this child’s sex?” While we note the question does not explicitly ask for sex at birth, respondents were limited to a male/female binary option limiting our results from reflecting gender diversity among autistic youth and/or youth with ADHD.

## Conclusions

The present study identified heightened anxiety and/or depression among autistic youth and/or youth with ADHD age 12–17 who experienced bullying victimization using NSCH data from the five survey years of 2016–2020 (*n* = 71,973). Remarkably, the difference in prevalence rates of anxiety and depression among autistic youth with and without ADHD who are bullied is a three to nine-fold difference (75% versus 22% for anxiety, and 41.8% versus 4.8% for depression), which highlights the detrimental impacts that bullying victimization may have on mental health and wellbeing.

These results identify the need to improve primary prevention efforts to reduce stigma and bullying victimization among all students, especially those most vulnerable. Furthermore, findings highlight the need for future research to understand the cyclical relationship among bullying, mental health conditions, and autism and/or ADHD. Implications for practice include recognizing that females experience higher incidences of bullying and mental health conditions. Recommendations include exploring new means to stop the bullying cycle, from bullying and mental health screening and support to school-wide anti-stigma initiatives that position autism and ADHD from a strength-perspective.
